# The consumption pattern and perception of using artificial sweeteners among the public in Tabuk region, Saudi Arabia

**DOI:** 10.3389/fpubh.2023.1166868

**Published:** 2023-06-22

**Authors:** Sawsan A. S. Alharthi, Khulud Hassan A. Alaisayi, Lina Yousef S. Alalawi, Raniya Omar S. Alamri, Karema Abu-Elfotuh, Tahani S. Alenazi, Palanisamy Amirthalingam, Hassan A. H. Albariqi, Asmaa A. Mohammed, Norah Alsubayti, Ahmed M. E. Hamdan, Magy R. Kozman

**Affiliations:** ^1^Pharm D Program, Faculty of Pharmacy, University of Tabuk, Tabuk, Saudi Arabia; ^2^Clinical Pharmacy Department, Faculty of Pharmacy (Girls), Al-Azhar University, Cairo, Egypt; ^3^Department of Clinical Pharmacy, College of Pharmacy, King Khalid University, Abha, Saudi Arabia; ^4^Department of Pharmacy Practice, Faculty of Pharmacy, University of Tabuk, Tabuk, Saudi Arabia; ^5^Eradah Mental Health Complex, Tabuk, Saudi Arabia; ^6^Pharmacology and Toxicology Department, Faculty of Pharmacy (Girls), Al-Azhar University, Cairo, Egypt; ^7^King Salman Armed Forces Hospital, Tabuk, Saudi Arabia; ^8^Clinical Pharmacy Department, Faculty of Pharmacy, Misr University for Science and Technology, Giza, Egypt

**Keywords:** artificial sweeteners, aspartame, consumer perception, food safety, steviana, weight control

## Abstract

**Background:**

Obesity and weight gain have become major problems worldwide. Thus, several forms of alternative intense sweeteners are extensively used, offering a non-caloric sweet taste. To the best of our knowledge, no research has studied either the consumption pattern or the perception of using artificial sweeteners in Saudi Arabia.

**Objectives:**

Our research aimed to study the usage pattern of such artificial sweeteners in the Tabuk region and estimate the knowledge of and attitudes toward their usage among the population.

**Methods:**

A cross-sectional study promoted on multiple social media platforms and face-to-face interviews in different malls and hospitals in the Tabuk region. We grouped the participants into two major groups: the users and the non-users of artificial sweeteners. Each group has been subdivided into a healthy subgroup and those with a medical record subgroup. Participants’ characteristics and their choice of sweeteners were analyzed using bivariate analysis. The age, gender, and education level of the participants were adjusted using binary logistic regression in order to adjust for potential confounders.

**Results:**

A total of 2,760 participants were included in our study. We found that more than 59% of the participants that were over 45 years old were non-hospitalized non-hospitalized diseased irrespective of their usage of artificial sweeteners. Furthermore, females, graduates, diabetics were significantly high irrespective of their subgroup. Moreover, Steviana^®^ is the most commonly used artificial sweetener. In addition, healthy participants showed a greater perception of the usage and adverse effects of artificial sweeteners. Furthermore, bivariate analysis using logistic regression revealed significant associations (*p* < 0.05) with confounders such as gender, age, and education level.

**Conclusion:**

Educational programs and nutritional advice for the safe consumption and the daily permissible doses of artificial sweeteners are essential and should be directed specifically at females.

## Highlights

- The majority of non-hospitalized users of artificial sweeteners were female and obese.
- Steviana® is the most widely used artificial sweetener.
- Users of artificial sweeteners showed higher proportions of common medical conditions than the non-users of artificial sweeteners.
- Healthy participants had a higher perception than the diseased population of artificial sweeteners.

## 1. Introduction

Weight gain and obesity are major problems worldwide, causing many non-communicable diseases such as cardiovascular diseases, cancer, kidney problems, and diabetes (1, 2). Weight and its impact on health are consistently found to be the top concerns in surveys. Accordingly, it is not surprising that most people are either trying to maintain or lose weight (3). A tapered treatment pyramid of obesity interventions includes nutritional selection, diet and/or calorie restriction, physical exercise, cognitive behavioral strategies, pharmacology, and surgery (3, 4). Globally, there are several weight loss drugs available, including probiotics and prebiotics, that are being considered as potential anti-obesity treatments (5). Some probiotic strains are shown to be effective in reducing body mass index (BMI) and hip circumference (6). In spite of these efforts, obesity continues to increase and has reached epidemic proportions. Today, the major goal of weight control, obesity control, and diabetes management is the control of blood glucose levels through refraining from sugars and replacing them with non-calorigenic sugar substitutes (7–9). The currently approved non-calorigenic sweeteners in the United States are aspartame, acesulfame-K, neotame, saccharin, sucralose, cyclamate, and alitame (7). Yet, these potent calorie-free sweeteners cannot simply substitute for such calorigenic sugars because the extent, quality, strength, and physical characteristics of such substitutes have not been determined yet. For example, some artificial sweeteners can give an intensely sweet taste. So, one tablespoonful of calorigenic sugar cannot be replaced by one tablet or one tablespoonful of an artificial sweetener. Moreover, the solubility and/or stability of the artificial sweeteners may hinder the usage of some artificial sweeteners to replace calorigenic sugars in some juices and foods. Besides, scientists and society still dispute the safety and health benefits of the consumption of these calorie-free sweeteners. Moreover, studies show that artificial sweeteners deteriorate the intestinal flora, adversely affect appetite control, and may have a lethal impact on probiotics (10). Still, the maximum allowable daily dose is a controversial topic (9). Recent population studies have suggested that long-term consumption of artificial sweeteners is significantly associated with adverse cardiovascular events—in particular, increasing coronary artery disease, stroke, and all-cause mortality (11). Notably, calorie-free sweeteners are metabolized in different ways owing to their versatile properties (12). Therefore, artificial sweeteners may have differences in metabolic rates, thereby explaining conflicting findings concerning a variety of biological mechanisms including body weight control and glucose homeostasis (13). Thus, it is not suitable to extrapolate the metabolic effects of 1 cal-free sweetener to other artificial sweeteners. In the same way, many studies have evaluated the metabolic fates of artificial sweeteners in rodents. Unfortunately, only a few researchers have studied the long-term effect of artificial sweeteners on humans (13, 14). Until now, most clinical studies on artificial sweeteners reported no associated beneficial effects on either body weight or glycemic control, although most studies were short-term. In this study, we studied the prevalence of consuming such sweeteners in the Tabuk region and estimated the knowledge of and attitudes toward their usage among the population in the Tabuk region.

## 2. Materials and methods

### 2.1. Sample size and sampling technique

The sample size was calculated using an online calculator; the total number is 799253; the official number of population in Tabuk region according to General Authority of Statistics in Saudi Arabia, the total number of population in Tabuk region (15), and the population proportion was 50%. Thus, the minimum sample size was 384 participants with a margin error of 9.6%.^1^

The sampling technique was a probabilistic type of technique that was done through both an online questionnaire spread through social media (Facebook, Twitter, etc.) and face-to-face interviews in malls, health centers, and hospitals.

### 2.2. Research design

A cross-sectional study was performed in the Tabuk region within a time period of 2 months, starting from October 1, 2022 to November 30, 2022. The study was approved by the Institutional Review Board (IRB) from the General Directorate of Health Affairs, Tabuk region (Registration no TU-077/022/165) and from the University of Tabuk (Registration no UT-231-83-2023). The participants had to be 18 years or older, of Saudi nationality, and internet literate to be eligible to take part in the survey through direct interview. Those who are living outside the city of Tabuk were excluded from the study. Before agreeing to participate in the survey, they were given access to an online information database. No personally identifying data (such as name, birth date, and email address) was saved.

A questionnaire was developed to measure the consumption of artificial sweeteners among the population. It has several parts including basic characteristics, details of consumption, response to reports, and perception of artificial sweeteners. The basic characteristics of the study participants were age, gender, education level, marital status, body mass index, physical activity, and smoking status. Relevant literature of studies measuring dietary habits among the population in the Tabuk region and studies measuring dietary habits among the Saudi nationals were consulted to identify the items to be included regarding the consumption of artificial sweeteners (16–20).

Christiansen et al. (21) developed and validated a community-based questionnaire that qualitatively assessed some items such as the customers’ perception of food additives including calorie-free sugars, believing information from regulatory bodies, and knowledge of regulations. We used this modified per-validated five-point Likert scale questionnaire (from strongly agree to strongly disagree which are represented by the scores 5–1, respectively) in order to estimate the participants’ perceptions of artificial sweeteners. The survey was translated by two bi-lingual academicians at the University of Tabuk. Moreover, a panel of experts comprising two healthcare professionals, one physician from the Department of Endocrinology and the other a pharmacist from the Department of Nutrition, evaluated and reviewed the questionnaire to determine the participants’ comprehension in terms of consumption details and perceptions of artificial sweeteners. A pilot study was used to understand the reliability of the questionnaire among 20 participants. The reliability statistics showed that the questionnaire has good internal consistency (Cronbach’s α > 0.8). In this survey, the term artificial sweeteners was used to refer to non-nutritive or calorie-free sweeteners. It is an understandable word used in the media and professional organizations. We used both English and Arabic versions of the survey.

**Figure 1:**
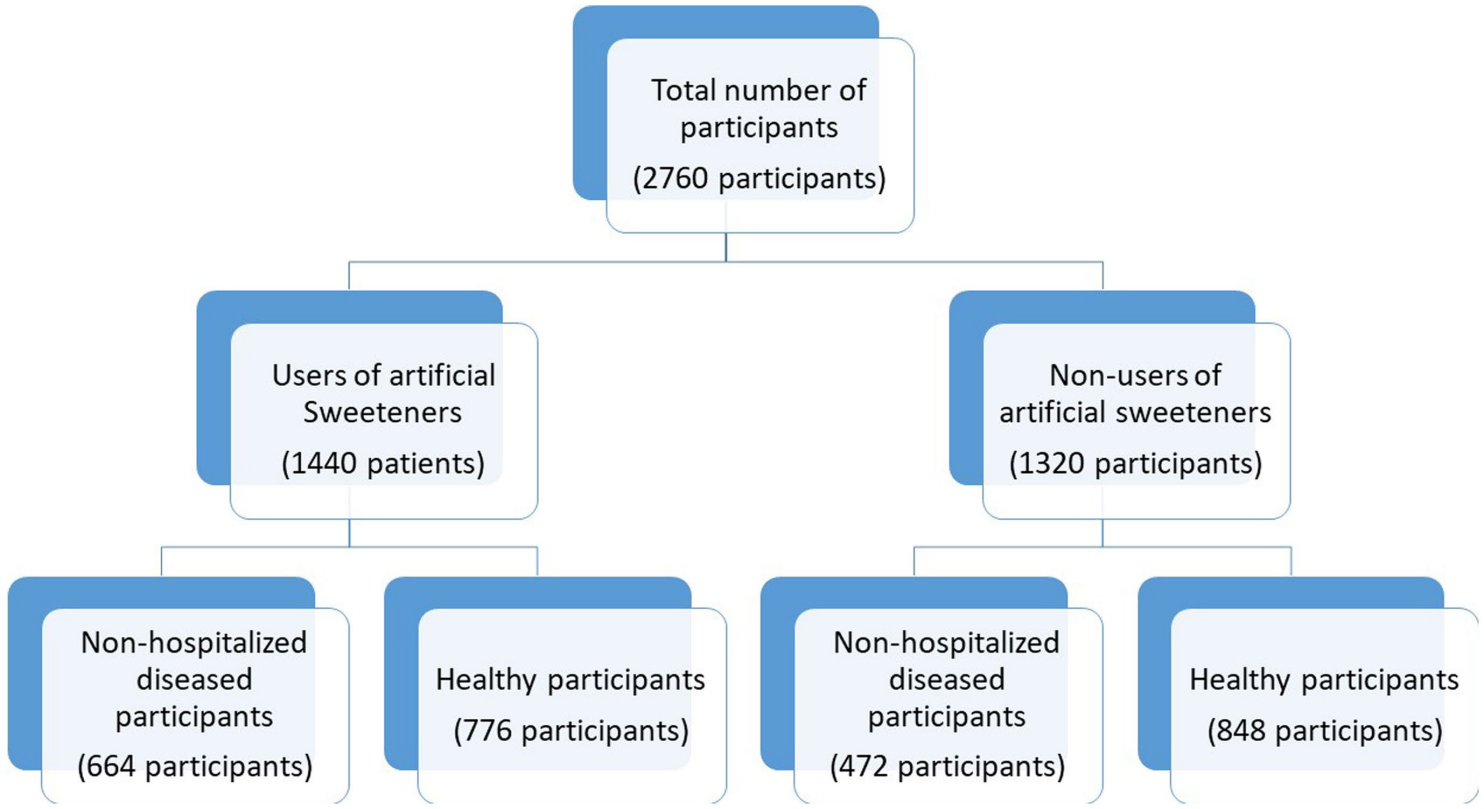
Grouping of the participants in the survey according to the usage of artificial sweeteners and their health conditions. ^*^The definitions of users or non-users are not technical terminologies. They are just descriptive terminologies for the consumers of artificial sweeteners.

The study was conducted through face-to-face interviews at different shopping malls in the city of Tabuk and promoted on multiple social media platforms (Facebook, Twitter) in order to facilitate sampling and to reach diverse users of online media in different parts of the Tabuk region with different educational backgrounds and occupations. The study participants were categorized initially into users and non-users of artificial sweeteners. Further, they were classified into non-hospitalized diseased and healthy participants (Figure 1). The participants with any current medical conditions and those who were free from any medical conditions were categorized as patients and healthy participants, respectively.

### 2.3. Statistical considerations

Statistical analysis was performed by using SPSS version 22. The items were coded. The results of the categorical variables were calculated as a number (percentage). The chi-square test or Fisher exact test was used to investigate the statistical difference between the patients and healthy participants. Mean (Standard deviation) was used to compare the perception of artificial sweeteners between patients and healthy participants. Binary logistic regression was performed to explore significant associations detected in the bi-variate analysis where adjusting for potential confounders such as age, gender, and education level was conducted. A value of *p* of 0.05 or less was designated as statistically significant for the applied statistical tests.

## 3. Results

As shown in Figure 1, in total, 2,760 study participants consented to participate in this cross-sectional survey (response rate of 69.42%). They were recruited based on the inclusion and exclusion criteria. In total, 52.17% (*n* = 1,440) were users of artificial sweeteners and 47.82% were non-users. Among the users, 46.11% (*n* = 664) were found to have medical diagnoses and were compared with the remaining 53.88% of healthy participants with no medical conditions (*n* = 776). Furthermore, a comparison was made between the healthy participants (*n* = 848; 64.24%) and patients (*n* = 472; 35.75%) among the non-users.

### 3.1. Basic characteristics of the participants in the survey

As shown in Table 1, the basic characteristics of the studied population were compared between the patients and healthy participants among both the users and non-users of artificial sweeteners. More than 59% of participants were older than 45 years among the patients, which was significantly higher than the corresponding healthy participants. Furthermore, participants were predominantly younger than 35 years old among both the users (*n* = 309; 41.1%; *p* = 0.000) and non-users (*n* = 320; 37.72%; *p* = 0.000) of artificial sweeteners. The characteristics of being a female, being a graduate, married and non-smoker status were significantly higher (*p* = 0.000) irrespective of the groups in this study. A significant proportion of the patients were overweight and obese compared with the corresponding healthy participants among both the users (*n* = 560; 84.33%; *p* = 0.000) and non-users (*n* = 368; 77.96%; *p* = 0.000) of artificial sweeteners. No significant difference between the patients and healthy study participants since the majority (65.5%) of them were physically active. Interestingly, the majority of users were not drinking sugar-free beverages and this difference was found to be statistically significant (*p* = 0.041). However, no significant difference (*p* = 0.201) was noted between the patients and healthy participants among the non-users of artificial sweeteners.

**Table 1:** Basic characteristics of study participants.

Variable	Users of artificial sweeteners (1,440)				Non-users of artificial sweeteners (1,320)			
	Non-hospitalized diseased (664)	Healthy (776)	Chi-square value	*p* value	Non-hospitalized diseased (472)	Healthy (848)	Chi-square value	*p* value
	*n* (%)	*n* (%)			*n* (%)	*n* (%)		
Age in years								
18–24	56 (8.43)	208 (26.80)	217.58	**0.000**	29 (6.14)	155 (18.27)		
25–34	40 (6.02)	111 (14.30)			35 (7.41)	165 (19.45)	137.40	**0.000**
35–44	171 (25.75)	247 (31.82)			128 (27.11)	232 (27.35)		
45–54	309 (46.53)	200 (25.77)			208 (44.06)	272 (32.07)		
≥ 55	88 (13.25)	8 (1.03)			72 (15.25)	24 (2.83)		
Gender								
Men	216 (32.53)	72 (9.27)	120.90	**0.000**	24 (5.08)	64 (7.54)	2.95	0.085
Female	448 (67.46)	704 (90.72)			448 (94.91)	784 (92.45)		
Education								
Illiterate	7 (1.05)	6 (0.77)	17.27	**0.000**	9 (1.9)	24 (2.83)	47.22	**0.000**
School	41 (6.17)	98 (12.62)			103 (21.82)	72 (8.49)		
Graduate	616 (92.77)	672 (86.59)			360 (76.27)	752 (88.67)		
Marital status								
Single	112 (16.86)	280 (36.08)	93.34	**0.000**	63 (13.34)	249 (29.36)	43.44	**0.000**
Married	469 (70.63)	451 (58.11)			369 (78.17)	535 (63.08)		
Divorcee	41 (6.17)	40 (5.15)			23 (4.87)	39 (4.59)		
Widow	42 (6.32)	5 (0.64)			17 (3.60)	25 (2.94)		
Body mass index								
Under weight (<18.5)	7 (1.05)	40 (5.15)	91.40	**0.000**	9 (1.90)	64 (7.54)	56.80	**0.000**
Normal (18.5–24.9)	97 (14.60)	253 (32.60)			95 (20.12)	288 (33.96)		
Overweight (25–29.9)	358 (53.91)	293 (37.75)			158 (33.47)	235 (27.71)		
Obese (≥ 30)	202 (30.42)	190 (24.48)			210 (44.49)	261 (30.77)		
Physical activity								
Yes	464 (69.87)	512 (65.97)	2.49	0.114	312 (66.10)	520 (61.32)	2.97	0.084
No	200 (30.12)	264 (34.02)			160 (33.89)	328 (38.67)		
Current smoker								
Yes	104 (15.66)	32 (4.12)	55.70	**0.000**	16 (3.38)	50 (5.89)	4.01	**0.045**
No	560 (84.33)	744 (95.87)			456 (96.61)	798 (94.10)		
Drinking sugar sugar-free beverages								
Yes	128 (19.27)	184 (23.71)	4.14	**0.041**	80 (16.94)	168 (19.81)		
No	536 (80.72)	592 (76.28)			392 (83.05)	680 (80.18)	1.62	0.201

* Chi-square analysis; *p* < 0.05 considered as statistically significant.

The bold values is for the significance. In order to grasp the attention of the reader.

### 3.2. Comparative analysis between the medical conditions of participants using artificial sweeteners and non-using artificial sweeteners

**Figure 2:**
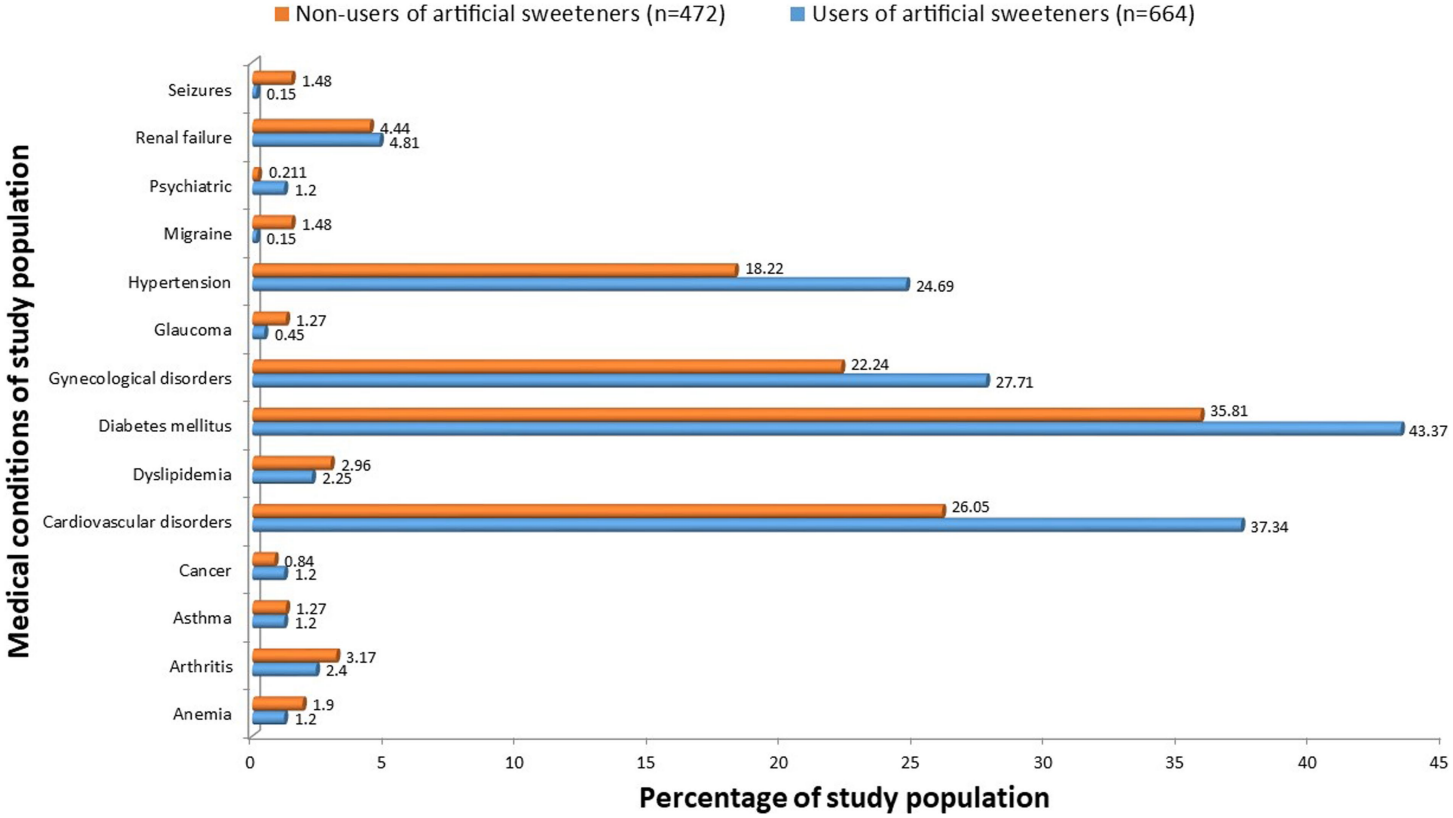
Comparison between medical conditions of users and non-users of artificial sweeteners.

As shown in Figure 2, a comparison was made regarding the medical conditions between the users and non-users. Diabetes mellitus was the leading medical condition in both the users (43.37%) and non-users (35.81%) followed by cardiovascular disorders, gynecological disorders, hypertension, and renal failure. Moreover, the proportions were higher among the users of artificial sweeteners compared with the non-users of artificial sweeteners.

### 3.3. Comparative analysis between detailed consumption of artificial sweeteners

As shown in Table 2, study participants significantly (*p* = 0.000) predominantly used one artificial sweetener and the frequency of consumption was once daily. The usage of Steviana® was significantly higher (*p* = 0.000) among the non-hospitalized diseased and healthy participants. A significant proportion (*p* = 0.000) of the participants in both subgroups had been using artificial sweeteners for less than a year. A higher number of participants were using artificial sweeteners in their hot drinks rather than baking. Both the above usages were found to be statistically significant. Significant proportions of patients and healthy participants reported that they were using artificial sweeteners to prevent or manage diabetes (*n* = 344; 51.80%) and obesity (*n* = 568; 73.19%), respectively.

**Table 2:** Details of consumption of artificial sweeteners.

Questions	Users of artificial sweeteners		Chi-square value	*p* value
	Non-hospitalized diseased (664)	Healthy (776)		
	*n* (%)	*n* (%)		
Frequency of consumption				
Once/day	296 (44.57)	368 (47.42)	38.41	**0.000**
Twice/day	232 (34.93)	208 (26.80)		
Three times/day	80 (12.04)	64 (8.24)		
More than three times/day	24 (3.61)	40 (5.15)		
Not specified/Unknown	32 (4.81)	96 (12.37)		
Number of artificial sweeteners using				
One	408 (61.44)	544 (70.10)	11.97	**0.000**
Two or more	256 (38.55)	232 (29.89)		
Types				
Candril®	16 (2.4)	5 (0.64)		
Steviana®	628 (94.57)	655 (84.40)		
Tropicana	188 (28.31)	207 (26.67)	42.22	**0.000**
Honey	148 (22.28)	229 (29.51)		
Not specified/Unknown	32 (4.81)	96 (12.37)		
Duration of use				
Less than 1 year	360 (54.21)	416 (53.60)	27.08	**0.000**
1 to <5 years	248 (37.34)	232 (29.89)		
5 to <10 years	8 (1.2)	8 (1.03)		
More than 10 years	8 (1.2)	24 (3.09)		
Not specified/Unknown	40 (6.02)	96 (12.37)		
Useing in hot drinks				
Yes	592 (89.15)	601 (77.44)	36.77	**0.000**
No	56 (8.43)	151 (19.45)		
Not specified	16 (2.4)	24 (3.09)		
Useing in baking				
Yes	256 (38.55)	240 (30.92)	9.21	**0.009**
No	384 (57.83)	504 (64.94)		
Not specified	24 (3.61)	32 (4.12)		
Reasons for using				
Prevent/manage diabetes	344 (51.80)	208 (26.80)	94.62	**0.000**
Prevent/manage obesity	320 (48.19)	568 (73.19)		

* Chi-square analysis; *p* < 0.05 considered as statistically significant.

The bold values is for the significance. In order to grasp the attention of the reader.

### 3.4. Comparative analysis between detailed consumption of artificial sweeteners

**Figure 3:**
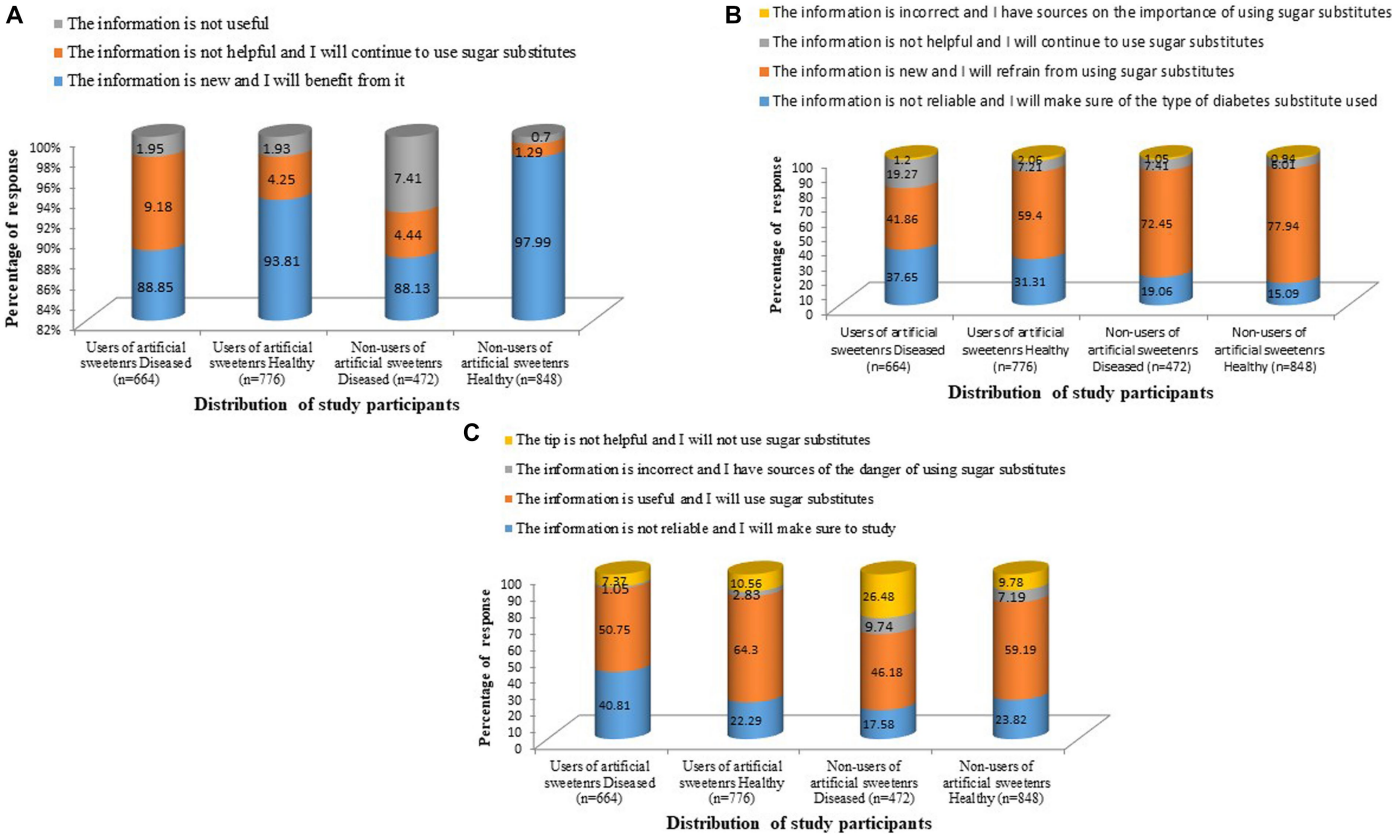
Response of the participants on social awareness of artificial sweeteners. On **(A)** “The Saudi Food Authority determined the level of sugar in the preparation.” **(B)** “Based on a study published in 2011 in the United States on experimental animals (22), it was proven that sugar alternatives may cause weight gain, colon cancer, brain cancer, bladder cancer, and some diseases.” **(C)** “Based on a study from the American Journal of Gastroenterology in 2020 (23), the study proved that by analyzing all the research published on sugar substitutes, they found that there is no association between sugar substitute.”

As shown in Table 3; Figure 3, the participants’ responses to a social awareness message from the Saudi Food and Drug Authority (SFDA) and recently published articles were noted. Regarding the level of sugar in the preparation of food, the participants predominantly found that the information was new and that they would benefit from it. Another social awareness message about sugar alternatives that may cause weight gain, cancer, and some diseases was shown to the participants and their response was obtained. The majority of the healthy participants responded positively (*n* = 461; 59.4%) as the information was new and they refrained from using sugar substitutes. However, the majority of the patients responded negatively to this information since they felt that the information was not reliable (*n* = 250; 37.5%) and they will make sure of sugar substitutes used. They further responded that the information was not helpful (*n* = 128; 19.27%) and that they would continue to use sugar substitutes. The difference between the responses of the patients and the healthy participants was statistically significant (*p* = 0.000). However, other published articles have concluded that there is no association between sugar substitutes and cancer and that they were very useful for improving cancer conditions. A significant proportion of the patients (*n* = 337; 50.75%) and healthy (*n* = 499; 64.3%) participants responded that the information was useful and they will use sugar substitutes. According to the participants’ responses, it was noted that social media was a significant informative resource in both the patients (*n* = 413; 62.19%) and healthy participants (*n* = 424; 54.63%).

**Table 3:** Participants’ response on social awareness of artificial sweeteners.

Awareness content	Types of response	Users of artificial sweeteners (1440)		Chi-square value^*^	*p* value
		Non-hospitalized diseased(664)	Healthy (776)		
		*n* (%)	*n* (%)		
The Saudi Food and Drug Authority determined the level of sugar in the preparation	The information is new and I will benefit from it	590 (88.85)	728 (93.81)	14.30	**0.000**
	The information is not helpful and I will continue to use sugar substitutes	61 (9.18)	33 (4.25)		
	The information is not useful	13 (1.95)	15 (1.93)		
Based on a study published in 2011 (22) in the United States on experimental animals, it was proven that sugar alternatives may cause weight gain, colon cancer, brain cancer, bladder cancer, and some diseases	The information is not reliable and I will make sure of the type of sugar substitute used	250 (37.65)	243 (31.31)	67.95	**0.000**
	The information is new and I will refrain from using sugar substitutes	278 (41.86)	461 (59.4)		
	The information is not helpful and I will continue to use sugar substitutes	128 (19.27)	56 (7.21)		
	The information is incorrect and I have sources on the importance of using sugar substitutes	8 (1.2)	16 (2.06)		
Based on a study from the American Journal of Gastroenterology in 2020 (23), the study proved that by analyzing all the research published on sugar substitutes, they found that there is no association between sugar substitutes and cancer, but rather that the use of sugar substitutes is very useful for improving the condition of cancers	The information is not reliable and I will make sure to study	271 (40.81)	173 (22.29)	60.75	**0.000**
	The information is useful and I will use sugar substitutes	337 (50.75)	499 (64.3)		
	The information is incorrect and I have sources of the danger of using sugar substitutes	7 (1.05)	22 (2.83)		
	The tip is not helpful and I will not use sugar substitutes	49 (7.37)	82 (10.56)		
Where do you get information regarding the benefits and safety of artificial sweeteners?	Social media	413 (62.19)	424 (54.63)	25.89	**0.000**
	Television/Radio	27 (4.06)	71 (9.14)		
	Reports and publications	133 (20.03)	201 (25.9)		
	Others	91 (13.70)	80 (10.30)		

* Chi-square analysis; *p* < 0.05 considered as statistically significant.

The bold values is for the significance. In order to grasp the attention of the reader.

As shown in Table 4, the perception of participants regarding the safety, benefits, and weight gain of artificial sweeteners was assessed using a Likert scale questionnaire. Both the non-hospitalized diseased and healthy participants thought that artificial sweeteners cause weight gain and have more benefits rather than risks. However, the perception of the safety of artificial sweeteners was found to be less prevalent. Moreover, there was no significant difference between the non-hospitalized diseased and healthy participants among the users. Similarly, the non-users for artificial sweeteners had not high perception regarding the written statements concerning the safety of artificial sweeteners or their claim to cause weight gain. However, healthy participants had significantly higher perception than the non-hospitalized diseased population towards the statements of the safety of the artificial sweeteners and their side effects of causing weight gain (*p* = 0.000). Besides, patients had significantly higher perceptions of the safety (*p* = 0.000) and benefits (*p* = 0.001) of artificial sweeteners than the risks regarding the usage of artificial sweeteners.

**Table 4:** Perceptions of study participants on artificial sweeteners.

Types of response	Users of artificial sweeteners (1440)		*p*	Non-users of artificial sweeteners (1320)		Value of *p*
	Non-hospitalized diseased (664)	Healthy (776)		Non-hospitalized diseased (472)	Healthy (−848)	
	Mean (SD)	Mean (SD)		Mean (SD)	Mean (SD)	
I think artificial sweeteners can cause weight gain	3.13 (0.88)	3.19 (1.00)	0.294	2.99 (0.90)	3.29 (0.82)	**0**
I believe that artificial sweeteners are completely safe for health	2.84 (0.76)	2.79 (0.90)	0.268	3.12 (0.80)	2.70 (0.88)	**0**
Artificial sweeteners have more benefits than risks for consumers	3.24 (0.83)	3.19 (0.91)	0.232	3.23 (1.06)	3.03 (0.99)	**0.001**

* Student-*t* analysis; *p* < 0.05 considered as statistically significant.

The bold values is for the significance. In order to grasp the attention of the reader.

### 3.5. Assessment of the association between the reason for using artificial sweeteners and using artificial sweeteners in hot drinks, with food, or in cooking according to gender and body mass index

As shown in Table 5, bivariate analyses using logistic regression produced statistically significant associations (*p* < 0.05) when confounders such as age, gender, and educational level were considered. Despite adjustments for age and education level, the odds ratios remained statistically significant for associations between artificial sweetener use and gender. This indicates that female perceptions of the usage of artificial sweeteners are an independent factor for developing disease since there is no influence on age and educational level. Additionally, when age, gender, and education level are adjusted, the association between artificial sweetener use in hot beverages, food, and cooking becomes statistically insignificant. In general, the usage of artificial sweeteners could be one of the factors associated with the development of diseases in the study population. However, this association has a possible influence on age, gender, and educational level.

**Table 5:** Logistic regression to explore the association between choices of sweeteners and soft drinks according to gender and BMI.

Associations [Reference group]	Unadjusted odds ratio	*p* value	Adjusted odds ratio	*p* value
Reason for using artificial sweeteners according to gender [Females]	3.1 [2.1–8.2]	**0.021**	4.6 [1.6–10.1]	0.002^*^
Using artificial sweeteners in hot drinks, with food, or in cooking [BMI less than 30]	1.6 [1.02–3.6]	**0.04**	1.5 [0.9–1.8]	0.09^**^

* Adjusting for age and education level.

** Adjusting for age, gender, and education level.

The bold values is for the significance. In order to grasp the attention of the reader.

## 4. Discussion

Due to how they are marketed, artificial sweeteners are considered to be healthy alternatives to calorigenic sugars which can provide the body with eatable food and palatable drinks (24). Many people use such food additives as substitutes for calorigenic sugars for weight loss and diabetes (25, 26). However, the beneficiary effect or the deleterious effect of these artificial sweeteners is controversial. Moon et al. (13) showed that the metabolism of artificial sweeteners is affected by the health of the body and the microflora of the intestine. Ruiz-Ojeda et al. critically discussed the evidence supporting the effects of artificial sweeteners, both synthetic (acesulfame K, aspartame, cyclamate, saccharin, neotame, advantame, and sucralose) and natural (thaumatin, steviol glucosides, monellin, neohesperidin dihydrochalcone, and glycyrrhizin) on the composition of microbiota in the human gut. Consuming acesulfame K for 4 weeks changes the population of the gut microbiota and decreases glucose fermentation by the cecal microbiota, suggesting that sweeteners affect glucose transport systems. Aspartame consumption for 11 weeks increases the fasting glucose concentrations and develops glucose intolerance. Furthermore, metabolomics analysis shows that aspartame was rapidly metabolized and related to short-chain fatty acids production, especially propionate production. Changes in the microbial composition were also observed in animals that received aspartame and the total bacteria and abundance of *Enterobacteriaceae* and *Clostridium leptum* increased. It is worth adding that artificial sweeteners interact with the T1R family of sweet-taste receptors in the oral cavity and gastrointestinal tract, and are thereby able to affect both appetite and satiety which can alter energy intake and body weight (14). Furthermore, several artificial sweeteners may reach the adipose tissue to interact with the T1R family of sweet-taste receptors and affect adipogenesis and, in turn, body weight.

Artificial sweeteners are present in the Saudi market in calorie-free beverages, candies, gums, chocolates, etc. Interestingly, some medications and OTC pharmaceutical products contain artificial sweeteners. Some artificial sweeteners are present in cold food and drinks. Others are included in hot drinks and baking (27). Such heating processes may lead to the decomposition and/or degradation of artificial sweeteners, leading to disturbance of the gut microbial flora and increasing the permeability of intestinal walls. This could result in both central and peripheral side effects (28). Some researchers have explored the beneficiary effect of artificial sweeteners for body weight control, glucose homeostasis, and underlying biological mechanisms (13). On the other side, it also has been proven that artificial sweeteners have deleterious effects on human health and caused weight gain, brain tumors, bladder cancer, and many other health hazards in animals (29–33).Thus, the safe daily dose of artificial sweeteners has to be extensively studied. According to the official government statistical reports for the classification of the prevalence of diabetes and obesity in the regions of Saudi Arabia, diabetes and obesity are the most prevalent in the Tabuk region. This indicates that there are nutritional and lifestyle problems in that region (15). Thus, we selectively chose this region for our study.

Our study targeted non-hospitalized participants in the Tabuk region, Saudi Arabia through a cross-sectional research design in order to investigate the consumption pattern and perception toward the usage of artificial sweeteners. However, we found that caloric sugar is still the most widely used sweetener. Furthermore, our research found that Steviana® is the most widely preferable artificial sweetener among the participants. It is a potent natural sweetener that is likely to become the major source of high-potency sweeteners (23). This result goes along with the previously published popularity of using stevia as an artificial sweetener in Japan (29). Stevia *rebaudiana* has been cultivated in Japan since the 1970s and Japanese people have widely used Steviana® for a long time in candies, chewing gums, bread, and pickle manufacturers (33). We found that Saudi Arabia is also now extensively using Steviana® as an artificial sweetener among non-hospitalized people especially in the absence of strong evidence to refute or support the safety of using artificial sweeteners. It may be due to the health perception in Saudi Arabia that stevia is a naturally derived artificial sweetener and that it may not have any adverse effects on human health. In our research, we found that females, graduates, non-smokers, patients with diabetes, and those who had a high body mass index (BMI) were those who were eager to highly consume artificial sweeteners generally (values of *p* < 0.05). Our results contradict previously published research for hospitalized patients in Saudi Arabia which reported that the same previously mentioned categories consume white sugar as a sweetener of choice (34). This contradiction in results may indicate that artificial sweeteners reduced the hospitalization of those categories and supports the hypothesis of the beneficiary effect of artificial sweeteners. Moreover, our research showed that the older the customer, the higher the consumption of artificial sweeteners. It has been reported that children and adolescents highly prefer artificial sweeteners due to their aim to control their weight and for prophylaxis against diabetes (35). We can explain such a contradiction that the national cancer institutes in both the United Kingdom and the United States are now supporting the use of artificial sweeteners in order to decrease the incidence of at least 13 kinds of cancers (36, 37). So, the age groups using artificial sweeteners have become older as these sites are highly visited by older aged adults. The national cancer institutes in the United Kingdom and the United States clearly advise people to use artificial sweeteners due to their proven safe use, although many researchers have proven the correlation between the usage of artificial sweeteners and many side effects (31, 38–41). They have not mentioned the safe daily dosage and the suitable drinks or foods; either cold, soft, hot etc. In 2013, the Saudi Health Interview Survey targeted 10,735 Saudis that were 15 years or older (42). Among the components measured in the study was Saudi dietary habits, measured via household visits. According to this study, Saudis consumed 115.5 mL of sugar-sweetened beverages on average per day. Moreover, men and younger ages consumed significantly higher amounts of sugar-sweetened beverages than females and children. This result may be due to the inclusion of both healthy and unhealthy participants in that survey. Although using artificial sweeteners can be a beneficial sweetening option for patients with diabetes and those with a high BMI, our research can raise an alarm about their usage and further investigations should be done for each disease separately. Interestingly, this study showed that regardless of the educational level of the participants, the population in the Tabuk region depends on social media more than any other sources to get information regarding the benefits and safety of artificial sweeteners. Finally, the population in the Tabuk region showed a willingness to benefit from information about artificial sweeteners and was ready to change their nutritional habits in order to keep them healthy.

## 5. Conclusion

The educational programs and nutritional advice for the safe consumption and the daily permissible doses of artificial sweeteners are essential and should be directed specifically at females.

## 6. Limitations of the study

There was no exact agreement among the participants regarding the amount of daily usage of such artificial sweeteners. Besides, the exact co-consumption of caloric sugars and their effect on body weight needed to be measured. Moreover, there are many medications in the Saudi market that also contains artificial sweeteners. These medications can be dispensed without a prescription. They are still not prohibited from usage by the Saudi Food and Drug Authority (Saudi FDA). Finally, the standard questionnaire, which was distributed through social media, did not give the space that was given through the direct face-to-face interview to follow the ideas of the non-users of the artificial sweeteners and the effect of this knowledge on their future prospects for starting consumption of artificial sweeteners.

## Data availability statement

The original contributions presented in the study are included in the article/supplementary material, further inquiries can be directed to the corresponding author.

## Ethics statement

The studies involving human participants were reviewed and approved by the Institutional Review Board (IRB) from the General Directorate of Health Affairs, Tabuk region (Registration no TU-077/022/165) and from the University of Tabuk (Registration no UT-231-83-2023). Written informed consent for participation was not required for this study in accordance with the national legislation and the institutional requirements.

## Author contributions

AH and KA-E: conceptualization. AH, SA, KA, LA, RA, PA, HA, and NA: data collection. AH and PA: drafting the work or revising it critically for important intellectual content. KA-E, AH, and PA: interpretation of data for the work. KA-E, AH, PA, AM, and MK: data analysis. All authors contributed to the article and approved the submitted version.

## Conflict of interest

The authors declare that the research was conducted in the absence of any commercial or financial relationships that could be construed as a potential conflict of interest.

## Publisher’s note

All claims expressed in this article are solely those of the authors and do not necessarily represent those of their affiliated organizations, or those of the publisher, the editors and the reviewers. Any product that may be evaluated in this article, or claim that may be made by its manufacturer, is not guaranteed or endorsed by the publisher.

## Footnotes

^1^https://www.calculator.net/sample-size-calculator.html?type=1&cl=95&ci=5&pp=50&ps=799253&x=66&y=18
